# Diabetic Lumbosacral Radiculoplexus Neuropathy as an Early Onset Postoperative Complication After Posterior Lumbar Fixation and Decompression

**DOI:** 10.7759/cureus.31625

**Published:** 2022-11-17

**Authors:** Konstantinos Zygogiannis, Spyridon I Antonopoulos, Ioannis Chatzikomninos, Savvas Moschos, Anastasios Kalampokis

**Affiliations:** 1 Trauma and Orthopaedics, Laiko General Hospital of Athens, Athens, GRC; 2 Orthopaedics, KAT General Hospital Attica, Athens, GRC; 3 Spine and Scoliosis Department, KAT General Hospital Attica, Athens, GRC; 4 Spine Department, KAT General Hospital Attica, Athens, GRC

**Keywords:** neurological complication, physical medicine and rehabilitation, neuro spine, diabete type 2, degenerative spine disease

## Abstract

Diabetic amyotrophy, also known as diabetic lumbosacral radiculoplexus neuropathy (DLRN), is a condition associated with sudden pain apparition and progressive distal extremities weakness leading to ambulatory status. A wide range of causes and pathologies may be involved, rendering the diagnosis challenging. Uncontrolled type 2 diabetes mellitus (T2DM) can be a trigger factor for such disorders. Here, we present the case of a 71-year-old patient with chronic left quadriceps weakness and atrophy, accompanied by radiculopathy, who underwent a single-level posterior lumbar fixation and decompression. The patient with a history of T2DM postoperatively showed immediate relief regarding the pain and started the rehabilitation protocol on the second post-op day. One month postoperatively, he presented with accusations of sensory impairment, motor weakness, and pain.

## Introduction

Amyotrophy is an asymmetric neuropathy that statistically more often affects patients with type II diabetes [1], though sometimes its cause may vary (non-diabetic radiculoplexus neuropathy). With pain being usually the first symptom, as this condition progresses, asymmetrical muscle weakness and sensory deficits appear, rendering the diagnosis challenging because the same clinical features and characteristics appear in a variety of syndromes [2]. Progressive aggravation of this syndrome may be present for a few months up to two years. Gradual recovery can be observed only after the acute phase, while the severity of the symptoms is a strong prognostic factor for the patient’s recovery. The anatomical and pathophysiologic mechanism of diabetic amyotrophy is not completely understood, but there is evidence of injury to the peripheral nerves, nerve roots, and lumbosacral plexus, with accompanying axonal degeneration, demyelination, inflammation, ischemia, and immune-mediated microvasculitis [3].

The aim of this report is to raise suspicion of rare but severe neurologic complications due to often associated pathological causes such as type 2 diabetes mellitus (T2DM) that could compromise a surgical intervention.

## Case presentation

A 71-year-old patient with a history of T2DM and asymptomatic cervical myelopathy presented to our department with a two-year left radiculopathy and left quadriceps weakness and atrophy (L3 root 3/5), with the MRI showing a large herniated disc with central and foraminal stenosis causing significant compression. Taking into consideration the failure of conservative treatment for two months with oral medication and physical therapy, with the main criterion being the pain, the patient underwent a single-level posterior fixation and decompression. The patient followed a normal postoperative period, with remarkable improvement in pain, starting the rehabilitation protocol on the second postoperative day.

**Figure 1:**
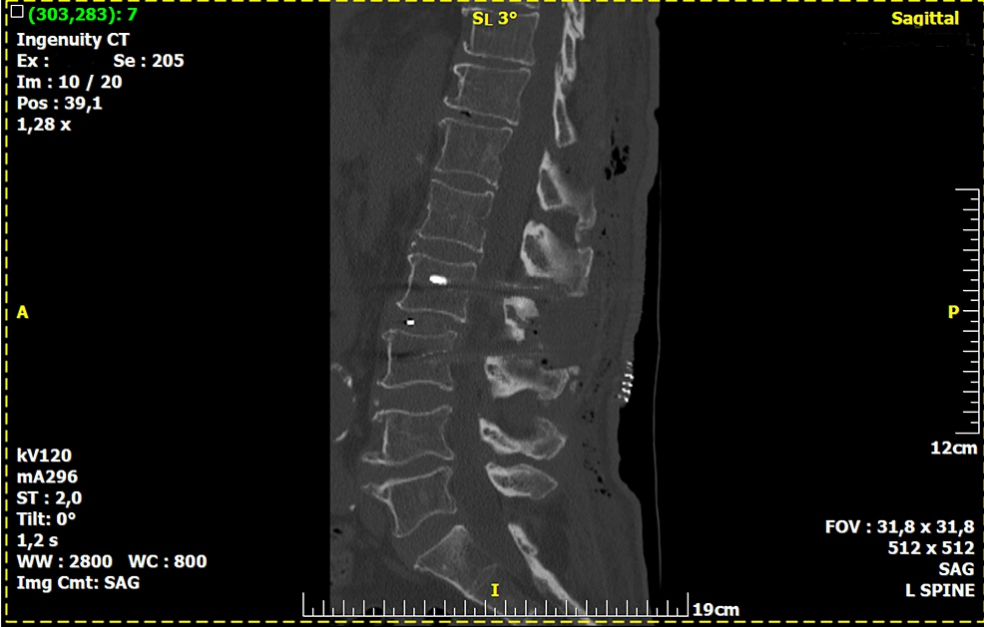
Postoperative CT scan of the lumbar spine Saggital view of CT scan showing satisfactory spinal alignment

**Figure 2:**
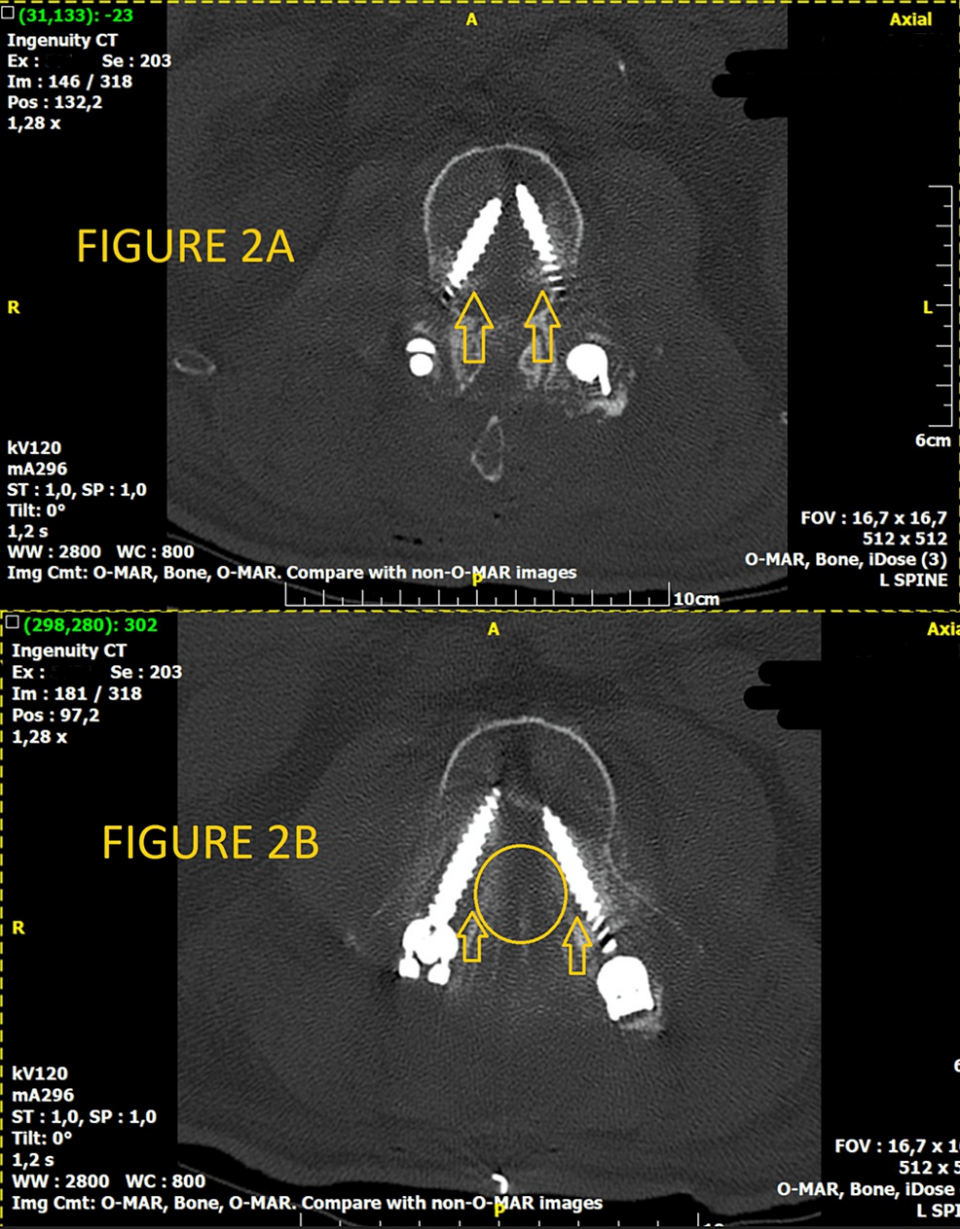
(A) L3 axial view of CT scan; (B) L2 axial view of CT scan Axial view of CT scan with the single-level screw fixation with no mechanical obstruction or cord perforation

**Figure 3:**
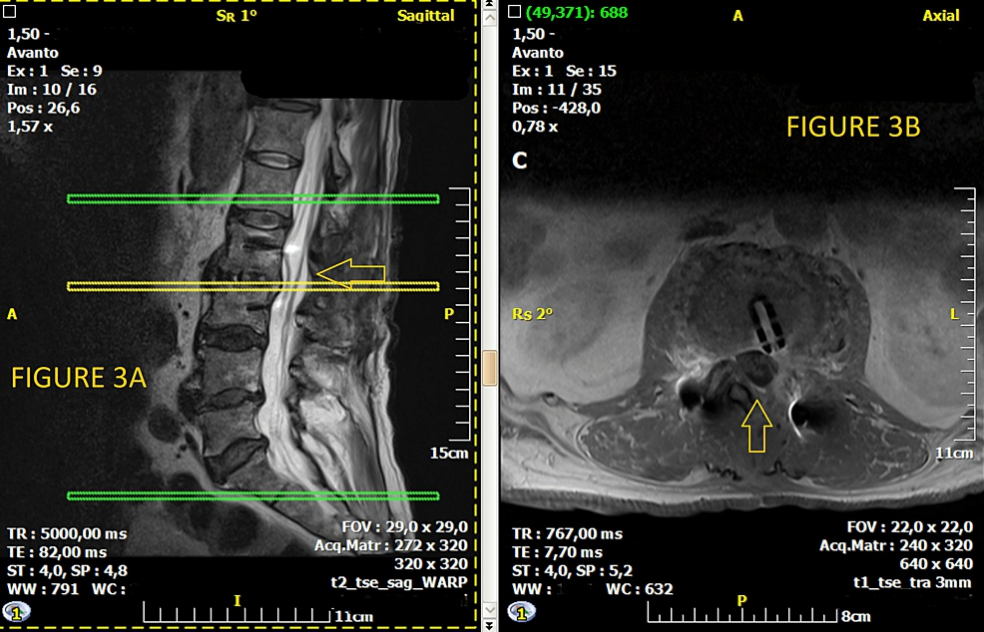
(A) At the left sagittal postoperative view of MRI scan; (B) at the right axial postoperative view of MRI scan MRI scan of the lumbar spine with no evidence of significant spinal cord compression

**Figure 4:**
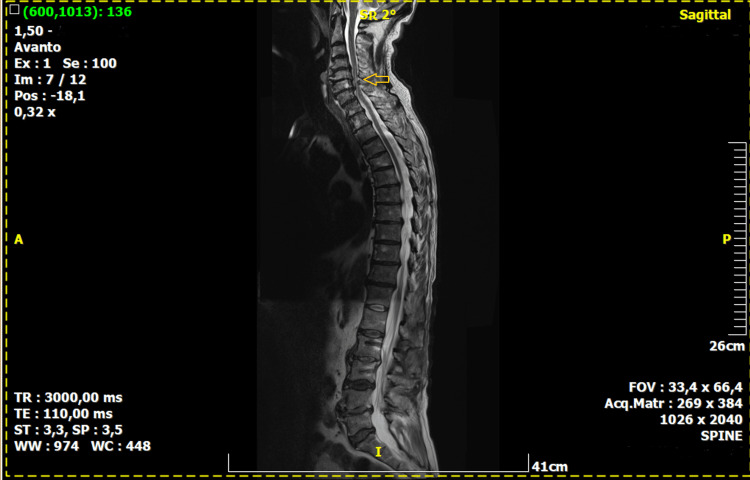
Full-body MRI scan Full-body MRI scan of the patient with the presence of asymptomatic cervical myelopathy

One month post-op, the patient came to the emergency department, presenting with pain and an inability to walk. The clinical examination initially revealed bilateral radicular pain with sensory impairment and L2, L3, and L4 bilateral roots affected; the upper extremities were unaffected. Blood results showed poor control of glucose levels (480 mg/dL). The posterior screw fixation was investigated with a CT scan (Figures 1-2) with the MRI scan unrevealing any spinal cord pathology (Figures 3-4). Ultrasound for the lower extremities also did not reveal any serious pathology. Excluding any other causes, electromyography in conjunction with the glucose blood levels and the clinical image supported the diagnosis of hyperglycemia-induced diabetic amyotrophy.

## Discussion

First described by Burns in 1890 and later defined by Garland in 1955 [4], diabetic amyotrophy is statistically found in less than 1% of patients with T2DM, affecting males more frequently than females. Although the pathogenesis of this particular syndrome is not well understood, with the two main theories regarding the causes being metabolic or ischemic, recent studies tend to support the ischemic cause. Patients with longstanding and poorly controlled diabetes can suffer from secondary metabolic derangements such as acceleration of nonenzymatic glycosylation [5] and activation of the sorbitol pathway [6].

Diabetic lumbosacral radiculoplexus neuropathy (DLRN) is characterized as a progressive condition until eventually it is stabilized. Gradual recovery can be expected when glucose levels return to normal levels in combination with no further clinical deterioration. In most case reports, the functional recovery is significant but often not complete. The severity of the symptoms and the glucose levels can affect the final rehabilitation result, which includes strict motor activity, muscle strengthening, and pharmaceutical therapy to control diabetes. Casey and Harrison [7], in a series of 12 patients, reported that only three had fully recovered, while in five cases there was significant residual motor impairment. Coppack and Watkins also reported that 26 patients diagnosed with diabetic amyotrophy showed no further clinical improvement over the course of 18 months [8].

Our patient remained at our department for two weeks until no further clinical deterioration was observed and the glucose levels settled back to normal values. Further, he was transferred to the department of physical rehabilitation, where his total treatment lasted for four months. Since L2, L3, and L4 roots at the time of transfer were 1/5 bilateral, his recovery was significant but not complete. At a one-year follow-up, with pain being absent, the patient is self-sufficient, with L2-L3 roots having 4/5 muscle strength and L4 roots having 3/5.

## Conclusions

DLRN is a serious clinical entity that should be taken into consideration as a rare postoperative complication for the whole spectrum of surgical operations. Patients with T2DM can often neglect their glucose levels, resulting in a wide range of conditions. In most cases where more than one lumbar root is affected and there is no mechanical cord compression, the differential diagnosis should include vascular and metabolic causes.
